# Classification and influencing factors of parent activation among parents of preterm infants at a NICU in China: a latent profile analysis

**DOI:** 10.3389/fped.2026.1715509

**Published:** 2026-05-20

**Authors:** Yuanfei Wang, Hong Che, Fangyu Zhu, Yan Chen, Kairong Lv

**Affiliations:** Neonatal Intensive Care Unit, Sichuan Provincial People's Hospital, University of Electronic Science and Technology of China, Sichuan, Chengdu, China

**Keywords:** cross-sectional study, latent profile analysis, newborn, parent activation, preterm

## Abstract

**Objective:**

To investigate the current status of parental activation among parents of preterm infants in a Chinese NICU, identify latent profiles, and analyze influencing factors across different profile categories.

**Methods:**

A cross-sectional study was conducted at a tertiary hospital in China from February to August 2025. Preterm infants and their parents were enrolled. During weekly family-integrated care sessions or at discharge, parents completed a questionnaire consisting of a demographic information form, the Parent-Patient Activation Measure, the Premature Infant Care Competency Scale, and the Social Support Rating Scale. Mplus 8.3 was employed to identify distinct parental activation profiles. Univariate and multivariate logistic regression analyses were subsequently performed to examine influencing factors associated with each identified profile.

**Results:**

The Parent-Patient Activation Scale score was 42.11 (31.58, 51.32). Latent profile analysis revealed that parental activation could be categorized into three distinct subgroups: the “Low Parent-Patient Activation Group” (28.9%), the “Moderate Parent-Patient Activation-Low Health Efficacy Group” (55.4%), and the “High Parent-Patient Activation Group” (15.7%). Multivariate logistic regression analysis indicated that gestational age, monthly household income per capita, level of social support, and preterm infant parental care competence were significant influencing factors for the latent profile of parental activation in parents of preterm infants (all *p* < 0.05).

**Conclusion:**

This study identified three distinct profiles of parental activation among parents of preterm infants in this Chinese NICU. Key influencing factors included gestational age, monthly per capita household income, parental competency, and social support. These findings underscore the importance of early identification of these profiles and the need for tailored interventions to strengthen parents' beliefs, knowledge, skills, and confidence in managing their infant's health.

## Introduction

Preterm birth, defined as delivery before 37 weeks of gestation, is a frequent obstetric complication and a leading cause of perinatal and under-five mortality and disability (1, 2). Due to their severely immature organ systems, preterm infants are highly vulnerable to complications such as respiratory distress, thermoregulatory instability, immune deficiency, and long-term neurodevelopmental impairments, necessitating coordinated and sustained multidisciplinary care from admission through the post-discharge period (3).

Once a preterm infant is admitted to the neonatal intensive care unit (NICU), clinical care is predominantly delivered by nursing staff. While this approach ensures medical stability, it often limits opportunities for parents to engage in hands-on caregiving or acquire the essential knowledge and skills needed for daily care, feeding, and interventional practices (4). This challenge is further compounded by the fact that preterm birth is frequently a traumatic experience for parents. The unexpected arrival of an infant with multiple healthcare needs can lead to a range of difficulties, including psychological distress and challenges in adapting to their new parental roles in a timely manner (5). This state can result in a delay in their acceptance and fulfillment of parental roles (6). As a result, families may lack adequate preparedness to provide early care, and parents may struggle to manage the home care of their preterm infants (7). This situation tends to negatively affect the infant's recovery from illness, hinder normal growth and development, and significantly increase the likelihood of hospital readmission (8).

In response to these challenges, models such as Family Integrated Care (FIC) have been developed to provide structured support for parental involvement within the NICU (9–11). The notion of integrated parental involvement in neonatal care can be traced back to initiatives such as the mother-infant unit, which was introduced by Professor Levin in 1994 (12). Building upon such foundations, FIC was subsequently developed as a structured model for implementation within the NICU (13). Research has demonstrated that FIC contributes to improved infant outcomes and reduced parental stress and anxiety (4, 14–16). This model has been implemented in several countries, including Canada, Australia, New Zealand, and the United States (4). Although some nations have a well-established tradition of encouraging and supporting parents to be closely involved in the care of their infants, the majority of neonatal intensive care units in China still restrict parental access (17). Consequently, it creates a significant barrier to parental learning, depriving them of crucial opportunities to gain the knowledge and competencies essential for caring for their vulnerable newborns. Moreover, it robs both parents and the child of one another's company and can negatively affect bonding (10).

The concept of “activation” was initially proposed by Hibbard et al., referring to a patient's belief, knowledge, ability, and confidence in self-health management (18). “Parent activation” is defined as the set of beliefs, knowledge, skills, and confidence that enables parents to manage their child's health, reflecting both their level of effective involvement and the practical role they play in the care process (19). Studies have shown that parents with high levels of activation are more likely to engage in their child's treatment and care process, thereby effectively improving health outcomes, enhancing satisfaction with healthcare services, reducing medical costs, and alleviating their own care-related psychological stress (20–22). Pennarola et al. found that children undergoing hematopoietic stem cell transplantation with highly activated parents exhibited a reduced incidence of infections, improved health status, and increased school attendance rates. Similarly, interventions providing health guidance to parents of medically complex children have been shown to increase parental activation (19). This heightened activation is correlated with a reduction in emergency department visits as well as an enhancement in the children's quality of life (23). Therefore, enhancing parental activation can be regarded as a key intervention strategy for improving child health outcomes and optimizing the use of healthcare resources.

In the domain of neonatal care, preterm infants generally lack the decision-making capacity, thus designating their parents as the primary responsible parties for their child's health. Thus, the enhancement of parental activation is pivotal in the transformation of parents' involvement from a potentially perceived state of passivity and ineffectiveness to one of active and effective participation in the care of their children (24). One study revealed that parents of NICU infants exhibited notably higher activation levels than those documented in studies of adult patients or other pediatric populations (21). However, researchers found that the level of parental activation among 62 preterm infant caregivers was similar to that measured in parents of children with autism (25). Notably, following intervention through optimized patient- and family-centered care measures, parental activation among preterm infant caregivers increased significantly (26). Overall, these findings indicate that research on parental activation in neonatal and preterm care settings remains limited and requires further investigation.

Although FIC has been widely implemented in NICUs globally, clinical practice still largely adheres to a relatively standardized approach. While this structural strategy has yielded significant benefits, it has not adequately addressed substantial heterogeneity among families in terms of “parent activation” levels. Identifying such heterogeneity requires a method capable of revealing latent subgroups based on multidimensional observed indicators. Latent Profile Analysis (LPA), as a person-centered statistical method, can identify unobserved heterogeneous subgroups within a population based on individuals' responses to a set of observed variables (27). Accordingly, within the context where family-centered care has become the standard model, this study does not aim to re-establish the importance of parental involvement. Instead, this study aims to address this critical gap by employing LPA to: (1) identify and classify distinct profiles of parent activation among parents of preterm infants in the NICU, thereby moving beyond the traditional perspective; and (2) explore factors associated with membership in each profile, thereby providing empirical evidence to inform the development of stratified and more tailored family support strategies.

## Method

### Study design and participants

This study employed a cross-sectional design and enrolled premature infants hospitalized in the NICU of a tertiary hospital in China between February and August 2025. Their parents were also recruited based on predefined inclusion and exclusion criteria. Inclusion criteria were as follows: (1) infants with a gestational age below 37 weeks who were admitted to the NICU directly after birth; (2) parents aged 18 years or older who could understand the study materials; and (3) parents who were informed about the study, voluntarily agreed to participate, and provided written informed consent. Exclusion criteria were: (1) premature infants who died during treatment or for whom treatment was discontinued following a formal request; and (2) Parents with major psychosis formally diagnosed by a physician (e.g., schizophrenia, bipolar disorder with psychotic features). The study protocol was approved by the Ethics Committee of Sichuan Provincial People's Hospital for Basic and Clinical Research (Ethics Approval No. 2025273) and was conducted in accordance with the principles of the Declaration of Helsinki.

### Procedures

Following the initial NICU admission of preterm infants, researchers performed preliminary screening via the electronic medical record system based on medical diagnosis, parental age, and gestational age. Eligible infants who met the inclusion criteria were enrolled, and questionnaires were distributed during weekly family-integrated care sessions or upon discharge. Prior to data collection, all participants were fully informed of the study objectives and questionnaire details, and written informed consent was obtained.

### Measures

#### General information questionnaire

The research team developed the general information questionnaire, which included parental age, residential address, monthly per capita household income (yuan), educational background, mode of delivery, the infant's gender, number of other living children, the infant's birth weight, and gestational age.

#### Parent-patient activation measure

The Parent-Patient Activation Measure (P-PAM) was derived from the original PAM through a collaborative adaptation by Pennarola et al. and Hibbard (19). It has been validated and demonstrates a strong correlation with the original PAM, as well as acceptable internal consistency. The Chinese version of the scale used in this study was translated and culturally adapted by Zou for application in pediatric care settings in China (26). The P-PAM uses a 4-point Likert scale ranging from 1 “strongly disagree” to 4 “strongly agree”. Any items that the caregiver deems inapplicable to the child's condition receive a score of 0, with higher total scores reflecting greater caregiver activation. In this study, the Cronbach's alpha is 0.892.

Hibbard defined four levels of patient activation based on a 0–100 standardized score: (1) Level 1 (≤47.0): Caregivers show limited awareness of their child's health management and passively follow treatments without seeing themselves as active participants. (2) Level 2 (47.1–55.1): Caregivers recognize the value of involvement but lack sufficient knowledge and skills for effective management. (3) Level 3 (55.2–67.0): Caregivers adopt healthier behaviours and gain confidence, though they may still need support during stress or new challenges. (4) Level 4 (>67.1): Caregivers demonstrate strong knowledge, confidence, and the ability to sustain management under stress.

#### Premature infant care competency scale

The Premature Infant Care Competency Scale (PICCS) for parents of preterm infants during the transitional period includes four dimensions: care knowledge, care attitude, care skills, and social support, totaling 35 items (28). The Chinese version of PICCS was developed by Zhao (29). The self-administered scale adopts a Likert 5-point scoring method, ranging from “completely unaware or unmastered” to “completely aware or mastered,” corresponding to scores of 1–5, respectively. The total score ranges from 35 to 175, with higher scores indicating stronger parental care competence.

#### Social support rating scale

The Social Support Rating Scale (SSRS), developed by Xiao, is used to assess the level of social support among parents of preterm infants (30). It consists of 10 items across three dimensions: subjective support, objective support, and utilization of support. The scale employs a multi-dimensional scoring method, with a total score of 66. Higher scores indicate greater perceived social support. Support levels are categorized according to total scores: a score below 35 denotes low support, 35–45 moderate support, and above 45 as high support.

### Statistical analysis

The latent profile was conducted by Mplus 8.3 Software; starting with one-category model, gradually increasing the number of potential profiles, testing the adaptability of each model, and ultimately selecting the best potential profile model. The assessment of the optimal latent profile was based on fit indices including the Akaike Information Criterion (AIC), the Bayesian Information Criterion (BIC), and the sample-size adjusted BIC (aBIC). Lower values on these criteria indicate a better model fit. Classification accuracy was evaluated using entropy, which ranges from 0 to 1, when Entropy ≥0.8, it shows that the current classification accuracy is more than 90%, and with values closer to 1 reflecting higher precision in profile classification. The Lo–Mendell–Rubin (LMR) test and the Bootstrap Likelihood Ratio Test (BLRT) were used to compare the fit between models with varying numbers of latent categories. A significant *p*-value (*p* < 0.05) for either test suggests that the model with k categories provides a better fit than the model with k-1 categories.

After determining the optimal latent profile model, statistical analysis was performed using SPSS 26.0. Continuous data were described using mean and standard deviation or median and interquartile range (IQR), while categorical data were summarized with frequencies and percentages. Using the identified latent profile categories as the dependent variable, chi-square tests were applied for categorical data, and the nonparametric Kruskal–Wallis test was used for continuous data. Additionally, multivariate logistic regression was employed to analyze influencing factors, with a statistical significance level set at *p* < 0.05.

## Result

### General characteristics of parent and preterm infants

In total, 255 questionnaires were distributed and collected in this study. Among them, 6 invalid questionnaires were excluded, leaving a final sample of 249 parental participants. Of these participants, 139 (55.8%) were fathers and 110 (44.2%) were mothers. The mean age of fathers was 28.69 (±6.03) years, and the mean age of mothers was 28.27 (±5.18) years. Approximately 59.8% of the families had no other children, while 40.2% had more than one child. A total of 144 mothers (57.8%) delivered via cesarean section, and 105 (42.2%) had vaginal deliveries. Among the preterm infants, 114 were male (45.8%) and 135 were female (54.2%). The average birth weight was 2120.64 (±649.43) g, and the mean gestational age was 33.48 (±2.98) weeks.

### Parent-patient activation measure level

After standardizing the total P-PAM score, the parental activation score for parents of preterm infants was 42.11 (31.58, 51.32). The parental activation levels were divided into four levels: the first level (≤47) included 160 individuals (64.3%), the second level (47.1–55.1) included 34 individuals (13.7%), the third level (55.2–67.0 points) included 28 individuals (11.2%), and the fourth level (>67.1) included 27 individuals (10.8%). P-PAM median score of mothers was 42.11, and for fathers was 39.47.

### Subgroups identified by LPA

A latent profile analysis was conducted on the 13 items of the P-PAM. Starting with an initial one-class model, the number of classes was incrementally increased. As the number of classes increased, the values of AIC, BIC, and aBIC gradually decreased. When a three-class model was retained, the rate of decrease in the AIC, BIC, and aBIC values began to plateau. The entropy value was 0.885, and both the LMR and BLRT tests were statistically significant (*p* < 0.05). Comprehensively considering all statistical indicators, practical significance and interpretability, the three-class model was identified as the optimal fitting model (Table 1).

**Table 1:** Fitness indicators of different latent profiles.

Class	AIC	BIC	aBIC	LMR *p* value	BLRT *p* value	Entropy	Proportion (%)
1	7785.011	7876.465	7794.044				
2	7038.197	7178.895	7052.093	<0.001	<0.001	0.944	79.9/20.1
**3**	**6793** **.** **815**	**6983** **.** **757**	**6812** **.** **574**	**<0** **.** **001**	**<0** **.** **001**	**0** **.** **885**	**28.9/55.4/15.7**
4	6697.645	6936.832	6721.268	0.028	<0.001	0.859	22.1/36.1/26.5/15.3
5	6626.997	6915.429	6655.484	0.467	<0.001	0.869	25.3/23.7/32.1/7.2/11.7

AIC, Akaike information criterion; BIC, Bayesian information criterion; LMR, Lo–Mendell–Rubin adjusted likelihood ratio test; BLRT, bootrapped likelihood ratio test. Bold row was the chosen model.

**Figure 1:**
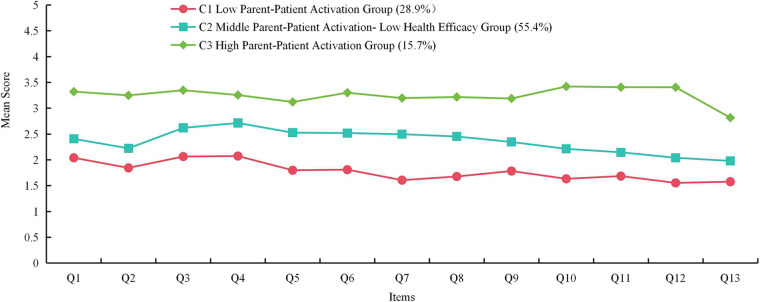
The characteristic distribution of three latent profiles of parent-patient activation.

Through latent profile analysis of parental activation among parents of preterm infants, the characteristics of three distinct latent profiles were identified and named based on their characteristics. As illustrated in Figure 1, C1 group scored the lowest on both the total scale and individual items, and was therefore designated as the “Low Parent-Patient Activation Group” (72 cases, 28.9%). The C2 group demonstrated moderate scores across both the total and individual items, but exhibited the lowest score specifically on item 2: “Taking an active role in my child's health is the most important thing that affects his/her health.” This group was classified as the “Middle Parent-Patient Activation–Low Health Efficacy Group” (138 cases, 55.4%). The C3 group achieved the highest scores and was named the “High Parent-Patient Activation Group” (39 cases, 15.7%).

### Univariate analysis of latent profiles on parent-patient activation

Parental activation scores were significantly lower in Group C1 (25.0; IQR 18.4–28.9) than in Group C3 (71.1; IQR 65.8–78.9) and Group C2 (44.7; IQR 39.5–50.0). Significant differences were also found among the three profiles in several key variables, including parental age, per capita monthly income, gestational age, preterm infant care competence, and social support (all *p* < 0.05). A detailed summary of these results is presented in Table 2.

**Table 2:** Univariate analysis of latent profiles on parent-patient activation (*n* = 249).

Variables	C1 (*n* = 72) Mean ± SD, *n* (%)	C2 (*n* = 138) Mean ± SD, *n* (%)	C3 (*n* = 39) Mean ± SD, *n* (%)	Test statistics	*p*
Age (Father)(years)	28.60 ± 6.11	27.91 ± 5.23	30.44 ± 5.90	3.100	0.047
Age (Mother)(years)	25.69 ± 5.02	26.06 ± 3.94	27.79 ± 4.93	3.057	0.049
Family residence					
Rural	34 (47.2%)	78 (56.5%)	15 (38.5%)	5.447	0.103
Urban/Town	38 (52.8%)	60 (43.5%)	24 (61.5%)		
Monthly income per capita					
≤2000	17 (23.6%)	29 (21.0%)	2 (5.1%)		
2001-4000	28 (38.9%)	36 (26.1%)	12 (38.8%)	15.468	0.017
4001-6000	13 (18.1%)	35 (25.4%)	7 (17.9%)		
>6000	14 (19.4%)	38 (27.5%)	18 (46.2%)		
Educational background (Father)					
< High school	4 (5.6%)	14 (10.1%)	7 (17.9%)	4.306	0.116
≥ High school	68 (94.4%)	124 (89.9%)	32 (82.1%)		
Educational background (Mother)					
< High school	10 (13.9%)	28 (20.3%)	7 (17.9%)	1.310	0.519
≥ High school	62 (86.1%)	110 (79.7%)	32 (82.1%)		
Type of delivery					
Cesarean section	49 (68.1%)	71 (51.4%)	24 (61.5%)	5.611	0.060
Vaginal	23 (31.9%)	67 (48.6%)	15 (38.5%)		
Infant Gender					
Male	33 (45.8%)	64 (46.4%)	17 (43.6%)	0.095	0.953
Female	39 (54.2%)	74 (53.6%)	22 (56.4%)		
Living children					
0	41 (56.9%)	80 (58.0%)	28 (71.8%)	2.771	0.250
≥1	31 (43.1%)	58 (42.0%)	11 (28.2%)		
Birth weight (g)	2095.49 ± 700.51	2101.20 ± 644.68	2235.90 ± 566.62	0.728	0.484
Gestational age (weeks)	32.84 ± 3.28	33.51 ± 2.90	34.56 ± 2.32	4.329	0.014
PICCS	149.47 ± 11.60	155.37 ± 10.97	158.82 ± 6.60	11.687	<0.001
Care knowledge	55.57 ± 5.67	57.48 ± 5.18	59.13 ± 2.28	6.936	0.001
Care attitude	35.42 ± 2.37	35.98 ± 1.98	37.13 ± 1.84	8.571	<0.001
Care skills	46.74 ± 5.23	48.53 ± 4.48	49.31 ± 3.40	5.169	0.006
Social support	11.75 ± 1.98	13.38 ± 1.22	13.26 ± 1.46	28.916	<0.001
SSRS	23.78 ± 3.84	26.01 ± 3.98	27.59 ± 3.51	13.905	<0.001
Subjective support	8.60 ± 1.84	9.13 ± 2.27	10.10 ± 2.13	6.292	0.002
Objective support	7.63 ± 3.07	8.75 ± 2.35	9.23 ± 2.29	6.118	0.003
Utilization of social support	7.56 ± 1.74	8.12 ± 1.95	8.26 ± 1.77	2.698	0.069

SD, standard deviation.

### Multivariate logistic regression analyses of three latent profile of parent-patient activation

Latent profiles of parental activation were included as the dependent variable, while indicators with statistical significance in the univariate analysis were taken as independent variables. Logistic regression analysis showed that care competency, social support and monthly income were potential influencing factors for parent-patient activation (*p* < 0.05), as shown in Table 3.

**Table 3:** Multivariate logistic regression analysis of latent profiles of parent-patient activation (*n* = 249).

Variable	C3 vs. C1				C2 vs. C1			
	*β*	*p*	OR	OR 95% CI	*β*	*p*	OR	OR 95% CI
Social support	0.244	<0.001	1.277	1.130–1.442	0.150	0.001	1.162	1.066–1.267
Premature infant care competency	0.147	<0.001	1.159	1.070–1.253	0.051	0.003	1.052	1.017–1.089
Gestational age	0.030	0.020	1.030	1.005–1.057	0.006	0.413	1.006	0.992–1.021
Age (Father)	0.026	0.640	1.026	0.922–1.142	−0.029	0.434	0.971	0.902–1.045
Age (Mother)	0.037	0.588	1.038	0.908–1.187	0.033	0.503	1.033	0.939–1.137
Monthly income per capita								
≤2000	−2.679	0.003	0.069	0.012–0.398	−0.649	0.182	0.523	0.202–1.356
2001–4000	−1.247	0.031	0.287	0.093–0.890	−0.990	0.042	0.406	0.171–0.969
4001–6000	−1.066	0.116	0.344	0.091–1.300	−0.125	0.800	0.883	0.336–2.321

Monthly income per capita: >6000 as reference group, C1, Low Parent-patient activation group; C2, Middle Parent-patient activation–low health efficacy group; C3, High parent-patient activation group; C1 as reference group; OR, odds ratio; CI, confidence interval.

Given the higher proportion of fathers in this study (55.8%), we further tested the interaction between gender and the aforementioned influencing factors to examine the interaction effect of gender. The results showed that when distinguishing between C3 group and C1 group, the interaction between gender and key influencing factors did not reach statistical significance (*β* = 0.106, *p* = 0.238). However, when distinguishing between the C2 group and C1 group, the interaction was significant (*β* = 0.199, *p* < 0.001).

## Discussion

This study was carried out to identify distinct profiles of parental activation among NICU preterm infants' parents and to explore associated factors. The study was conducted with 249 parents whose preterm neonates were hospitalized in the NICU. The proportion of fathers (58.8%) was higher than other studies (31, 32). This can be primarily attributed to the following contextual factors. In the Chinese cultural context, most postpartum mothers observe the traditional practice of “zuo yue zi” (confinement), during which they are required to stay at home for 28–42 days and are strictly advised to avoid going outdoors (33). Consequently, it was often the fathers who participated in the research during the period when their preterm infants remained hospitalized.

The findings of this study revealed a generally low level of parental activation among parents of premature infants in these Chinese NICU. Specifically, 64.3% of parents demonstrated limited understanding of their role in their infant's health management, passively adhered to prescribed treatments, and lacked a sense of active involvement in the care process. In contrast to the findings of Skeens et al. and Zou et al., the PAM scores in the present study were considerably lower (21, 26). This discrepancy may be partially attributed to structural factors associated with hospital visitation policies in China. The study was conducted in a provincial tertiary hospital, where the NICU implements a fixed weekly parental presence policy, with each session lasting approximately 30 min. During these designated visitation periods, parents are supported to enter the unit and engage in basic caregiving activities, including diaper changing, feeding, and kangaroo mother care. While this policy grants parents limited opportunities for hands-on caregiving experience, the stringent time constraints may objectively impede the continuous development of caregiving skills and the establishment of confidence in health management for their infants. Furthermore, a subset of participants did not attend any visitation sessions during their infant's hospitalization and only participated in the study at the time of discharge. We therefore identify these restrictive visitation policies as key structural factors contributing to the high proportion of parents with low activation observed in this study. It is reasonable to hypothesize that in hospitals with more flexible visitation policies, such as extended visitation hours or daily access, parents would have increased opportunities for caregiving practice (34), which might correspondingly reduce the proportion of parents with low activation. More importantly, within the ethos of family integrated care, which is practiced in most parts of the world, parents are not seen as visitors but rather are encouraged and supported to be involved 24/7 in the care of their infants (35, 36). Therefore, it is important that healthcare teams, administrators, and policy makers in China review current practice and, while remaining culturally sensitive, offer parents the choice of 24/7 involvement in their infant's care.

In this study, parents in the High-Activation groups tended to be older, their infants had greater gestational ages, and a larger proportion of families had a monthly income above 6,000 yuan. In the low activation group, the primary characteristics include younger parental age, lower gestational age of the infant, reduced monthly household income, and lower birth weight. A possible explanation is that younger parents in this group, often early in their careers, face greater financial constraints due to lower income levels. These pressures limit their capacity for self-education and result in insufficient understanding of preterm birth-related care (37). Following the preterm birth, they likely perceived their infant's condition as more severe and incurable, which contributed to elevated levels of stress (38).

Under the influence of China's family planning policy, only-child individuals now constitute the majority of the younger generation, leading to the increasingly prevalent “Sandwich Generation” phenomenon. While navigating professional challenges, many newly married couples also bear the responsibility of supporting four elderly parents from both families. Under such multifaceted pressures, a growing number of young individuals exhibit signs of emotional detachment (39). When a child is born preterm and immediately admitted to the NICU, this forced separation not only hinders the development of parental identity but also adversely affects the formation of secure attachment/bonding between parent and infant. This suggests that healthcare providers should take into full consideration the actual circumstances of parents and initiate health education through diverse forms and channels at an early stage to meet their learning needs. For instance, opportunities for parent-preterm infant contact (such as implementing family-integrated care) can be created as early as possible to help parents adapt to their roles gradually before the infant is discharged, alleviate emotional detachment, and enhance caregivers' confidence.

This study shows that lower gestational age in preterm infants is associated with lower levels of parental activation. This finding may be explained by two principal reasons. First, lower gestational age in preterm infants is generally associated with prolonged hospitalization, greater disease severity, poorer long-term prognosis, and higher risk of complications, thereby subjecting parents to multifaceted pressures—including psychological, physical, financial, and social burdens. Second, the degree of nervous system immaturity is inversely related to gestational age, which correspondingly increases the likelihood of future motor impairments and psychological-behavioural problems in the preterm infant (40). Therefore, it is recommended that healthcare professionals strengthen their attention and provide systematic support to parents of preterm infants with lower gestational age, especially those at risk of neurological developmental impairments. During hospitalization, parental visitation and accompanying time may be appropriately extended, and evidence-based interventions such as Kangaroo Mother Care should be actively promoted to support neurobehavioral development in preterm infants and improve their long-term neurological outcomes (41). Meanwhile, standardized health education and practical bedside care guidance should be provided to enhance parents' caregiving skills and confidence, thereby increasing their willingness and ability to participate in care. After discharge, parents should also be encouraged to consistently bring their children for regular outpatient follow-ups and neurobehavioral assessments, so as to facilitate early detection of developmental deviations, and enabling timely intervention.

“Premature Infant Care Competency” is a multidimensional and comprehensive concept that refers to the totality of knowledge, skills, confidence, and psychological adaptation required by parents or primary caregivers to prepare for the safe discharge of a premature infant from the hospital and subsequent successful home care (42). In this study, parental care competency was positively correlated with parent-patient activation, with each 1-unit increment associated with 15.9% increased odds of being in the high activation group. Parents with relatively weaker preterm infant care knowledge and skills are more likely to fall into the low and medium activation groups compared to those in the high activation group. The care competence for preterm infants during the transition period is grounded in four core components: sufficient knowledge and practical skills in neonatal care, attentiveness to infants' needs, problem-solving capabilities, and a sense of parental responsibility (43). Research indicates that the current caregiving capacity of most parents of preterm infants does not yet meet the demands of home-based care for these vulnerable infants (44). This global issue has been consistently documented across high-income and low- and middle-income countries (7, 45). In this study, most parents possessed at least a high school education, reflecting sound learning abilities and the capacity to utilize various resources (e.g., the internet and books) to obtain knowledge related to preterm infant care. They rated themselves as having a general understanding of preterm care knowledge, their competencies were sufficient only for basic daily care tasks-such as feeding, diaper changing, and skin cleaning. However, substantial gaps were identified in more specialized domains, including preterm infant feeding techniques, emergency management of choking or regurgitation, intervention for apnea, early growth and developmental intervention, post-discharge nutritional strategies (e.g., transition from preterm to term formula and use of human milk fortifiers), and medication administration (e.g., vitamin D_3_, iron, and calcium supplementation). These domains should be prioritized by healthcare providers in the context of parental health education.

This study demonstrates a positive correlation between social support and parental activation in parents of premature infants. Social support refers to the various forms of resources that individuals obtain through their social networks (such as family, friends, colleagues, community, etc.), which can promote their physical and mental health, enhance well-being, and help them cope with life stressors (46). Social support for parents of premature infants primarily comes from family support, particularly the companionship of family members. Preparing a stable home care environment can improve the parent-infant relationship, enhance their parenting efficacy and coping abilities, and enable parents to feel more confident in managing their child's physical and mental health issues (47). Furthermore, access to community resources enables parents of preterm infants to respond more effectively to life-threatening emergencies, thereby enhancing the quality of life for both the infant and parents. Evidence showed that early emotional support (such as care from partners, family, and friends) and informational support (clear, consistent guidance from healthcare providers) can help alleviate anxiety, feelings of helplessness, and post-traumatic stress symptoms in parents of premature infants (48, 49).

The Middle Parent-patient Activation-Low Health Efficacy Group is defined by a moderate level of parent-patient activation coupled with low health efficacy, which constitutes a contradictory psychological and behavioural state characterized by “possessing certain caregiving competencies while lacking sufficient confidence in health management practices”. This inherent imbalance between behavioural potential and cognitive appraisal renders individuals in this group more susceptible to fluctuations in their perceived caregiving capabilities and the perceived availability of external social support. Furthermore, the relationships between this profile and key influencing factors are more prone to being influenced by gender. In contrast, the High Parent-Patient Activation Group demonstrates a high degree of congruence between caregiving behaviours and health-related beliefs, wherein the level of caregiving activation is well-aligned with health efficacy, forming a stable psychological and behavioural pattern. Within this framework, the influence pathways through which key factors-including caregiving ability, social support, and monthly household income-impact group membership are relatively fixed and consistent. In summary, the gender plays a significant interaction role in distinguishing between the Middle Parent-Patient Activation-Low Health Efficacy Group and the Low Parent-Patient Activation Group. This finding not only holds statistical significance but also reveals the presence of gender specificity in the formation mechanisms of different care activation patterns, which needs further exploration as to the need for development of differentiated and targeted intervention strategies.

To our knowledge, this is the first study to adopt a latent profile approach to identify distinct profiles of parental activation among parents of preterm infants and to explore their associated influencing factors. Based on the characteristics of the three latent profiles, it is suggested that healthcare teams can provide stratified care tailored to different latent profiles of parental activation. For the low parent-patient activation group, healthcare providers should identify psychosocial needs, address work–family conflicts, and offer individualized health education, flexible family-integrated care, and sustained emotional support to alleviate coping difficulties and enhance activation. For the Middle Parent-Patient Activation-Low Health Efficacy group, medical staffs should strengthen psychological support and efficacy encouragement, using positive feedback, peer support, and scenario simulation to build caregiving confidence and resolve the state of “having ability but lacking confidence”. For the High Parent-Activation group, there is need to focus on stress coping by providing continuous healthy lifestyle guidance, stress management strategies, and phased goal-setting, while strengthening follow-up support to monitor implementation and prevent confidence depletion, thereby ensuring sustained high activation.

### Implications to practice

Since this is the first study of its kind to explore latent profiles of parental activation for parents of preterm infants in a Chinese NICU, it is important that further research is carried out in various neonatal settings and geographies to improve understanding of this domain. Healthcare providers in China could explore stratified care tailored to different latent profiles of parental activation. By identifying those risk factors associated with specific subgroups, including gestational age, monthly household income, parental competency in preterm infant care, and social support- providers can tailor their assessments and interventions to meet the unique needs of each parental profile. NICU nurses can create opportunities for parent-preterm infant contact as early as possible. This can be achieved by implementing models like FIC, which helps parents gradually adapt to their roles before the infant's discharge, alleviates emotional detachment, and enhances their caregiving confidence. When implementing FIC, NICU nurses should ensure that their educational content extends beyond basic daily care. It must specifically address the special needs of preterm infants. Furthermore, parents of preterm infants should be encouraged to attend regular outpatient follow-ups and neurobehavioral assessments for their children, with the dual purpose of enabling early detection of developmental deviations for timely intervention and monitoring the parents' own activation levels and caregiving competencies to provide necessary re-education and support.

### Limitations

There are some limitations in the study. First, all participants were recruited from a single provincial tertiary hospital in China, with a relatively small sample size. This restricted our ability to fully examine potential differences in parental activation patterns between fathers and mothers, as well as their associated influencing factors. Second, data were collected at a hospital with a fixed weekly visitation schedule allowing only 30 min per visit. It is therefore uncertain whether our findings can be generalized to hospitals with more open, flexible visitation policies or those with fully restricted access. Future research should incorporate samples from hospitals with diverse visitation policies to validate the cross-context robustness of our results. Furthermore, the study failed to distinguish clearly between primary and secondary caregivers, with all respondents categorized merely as parents. Since paternal and maternal caregivers can differ systematically in caregiving behaviours, interaction patterns and emotional expression, this undifferentiated grouping may compromise the precision of the research conclusions. Third, causality between the independent and dependent variables cannot be established due to the cross-sectional nature of this study. Additionally, this investigation was conducted exclusively at the time of discharge for premature infants, and therefore can only reflect parental activation status at that specific time point. It cannot capture the dynamic trajectory of how parental activation levels evolve throughout different care stages. Results might differ if data were collected at different time points, such as at birth or after the infant returned home. All the above-limit the generalizability of our findings.

## Conclusions and recommendations for further study and practice

This study revealed significant heterogeneity in parental activation levels among parents of preterm infants in a Chinese NICU. With LPA, three distinct subgroups were identified: “Low Parent–Patient Activation Group”, “Moderate Parent–Patient Activation–Low Health Efficacy Group”, and “High Parent–Patient Activation Group”. The findings indicated that gestational age, household monthly income, parental care competency, and social support were key influencing factors associated with parental activation.

If our findings are corroborated by further studies, healthcare providers could identify these profiles at an early stage, conduct targeted evaluations according to the characteristics of each subgroup, and deliver individualized interventions to strengthen parental beliefs, knowledge, skills, and confidence in managing their preterm infants' health.

Further multi-centre, large-sample longitudinal studies covering different geographies are needed to thoroughly investigate the typology of activation patterns among parents of preterm infants and to identify key influencing factors across various developmental phases.

## Data Availability

The original contributions presented in the study are included in the article/Supplementary Material, further inquiries can be directed to the corresponding authors.
